# Earlier Diagnosis of Alzheimer’s Disease by Dynamic Light Scattering Spectroscopy of the Eye

**DOI:** 10.1002/alz70861_108090

**Published:** 2025-12-23

**Authors:** Jeffrey Neill Weiss

**Affiliations:** ^1^ Parkland, FL USA

## Abstract

**Background:**

Dynamic Light Scattering (DLS) Spectroscopy measures changes in the Brownian movement of particles at the molecular level. Since the retina of the eye is a neural tissue and an outgrowth of the brain, a clinical instrument was developed capable of making DLS measurements from the retina in patients with mild cognitive impairment (MCI) undergoing PET nuclide imaging for the presence of cerebral amyloid.

**Method:**

16 patients with mild cognizant impairment (MCI) undergoing PET nuclide imaging for amyloid also underwent DLS testing in an IRB approved study.

**Result:**

10/16 (63%) patients tested positive for amyloid, and 6/16 (38%) tested negative. DLS testing correctly identified all 10/16 (100%) of the patients that were positive for amyloid, and 5/6 (83%) patients that were negative for amyloid. One patient exhibited a positive DLS measurement but a negative PET amyloid scan. In a prior study, the DLS measurement exhibited a predictive value, indicating that this patient will be diagnosed with Alzheimer’s disease by standard clinical methods in the future.

**Conclusion:**

The goal is to develop a treatment for Alzheimer’s disease early enough to prevent the development of symptoms. Dynamic Light Scattering Spectroscopy (DLS) offers the potential to make an earlier diagnosis of Alzheimer’s disease than with current methods. It is noninvasive, quantitative, inexpensive to perform, and should lead to the cost‐effective development of more effective pharmaceuticals.

